# Efficacy of Mongolian medicines warm acupuncture for knee osteoarthritis: a systematic review and meta-analysis

**DOI:** 10.3389/fmed.2026.1860943

**Published:** 2026-07-22

**Authors:** Risu Na, Qiqige Wulan, Hushan Ba, Wenxiang Guan, Lan Wu

**Affiliations:** 1 Affiliated Mongolian Medicine Clinical College of Inner Mongolia Medical University (International Mongolian Hospital of Inner Mongolia), Hohhot, Inner Mongolia, China; 2 Inner Mongolia Medical University, Hohhot, China

**Keywords:** complementary medicine, knee osteoarthritis, meta-analysis, Mongolian medicines warm acupuncture, pain management, systematic review, WOMAC scale

## Abstract

**Objective:**

This study aimed to systematically evaluate the efficacy of Mongolian medicines warm acupuncture for knee osteoarthritis (KOA).

**Methods:**

Eight databases, including China National Knowledge Infrastructure (CNKI), Wanfang, Chinese Science and Technology Periodical Database (VIP), Chinese Biomedical Literature Database (CBM), PubMed, the Web of Science, the Cochrane Library, and EMBASE, were searched from their inception to 31 December 2025 for randomized controlled trials (RCTs) evaluating Mongolian medicines warm acupuncture for KOA. The primary outcome was the clinical response rate (CR), whereas the secondary outcomes included the visual analog scale (VAS) and Western Ontario and McMaster Universities Osteoarthritis Index (WOMAC) scores. Risk of bias was assessed using the Cochrane risk of bias 2 (RoB 2) tool. Pooled effect sizes were calculated using RevMan 5.4 with fixed- or random-effects models. The certainty of evidence was graded using the Grading of Recommendations Assessment, Development, and Evaluation (GRADE) approach. The protocol was registered with the International Prospective Register of Systematic Reviews (PROSPERO) (CRD420261362311).

**Results:**

A total of 10 RCTs (*n* = 882) were included. In add-on design trials (2 studies, *n* = 120), the benefit was non-significant (risk ratio [RR] = 1.11, 95% confidence interval [CI]: 1.00–1.25; *p* = 0.06; *I*^2^ = 0%). In head-to-head comparisons (6 studies, *n* = 630), Mongolian medicines warm acupuncture significantly improved the clinical response rate compared with oral medications (RR = 1.25, 95%CI:1.17–1.33; *p* < 0.00001; *I*^2^ = 0%). Subgroup analysis by treatment duration showed no significant difference (P for subgroup difference = 0.55). Pain (VAS) was significantly reduced (3 trials; MD = –1.50, 95%CI:–1.99 to −1.01; *p* < 0.00001; *I*^2^ = 87%). WOMAC total score also preferred Mongolian medicines warm acupuncture (4 trials; MD = –12.45, 95%CI:–19.08 to −5.82; *p* = 0.0002; *I*^2^ = 95%). The certainty of evidence was moderate for the secondary analysis and low for the primary analysis, VAS, and WOMAC.

**Conclusion:**

Mongolian medicines warm acupuncture is effective in improving the clinical response rate and reducing pain in KOA when used as an alternative to oral medications. However, evidence from add-on design trials remains inconclusive due to the limited number of studies. High-quality, large-scale add-on RCTs are warranted.

**Systematic review registration:**

https://www.crd.york.ac.uk/PROSPERO/myprospero, Unique Identifier CRD420261362311.

## 1 Introduction

Knee osteoarthritis (KOA) is a common degenerative joint disorder characterized by progressive loss of cartilage, osteophyte formation, and narrowing of the joint space (1). Clinically, knee pain, stiffness, swelling, and restricted mobility are common, but in advanced osteoarthritis, deformities and disability can occur (2). KOA primarily affects middle-aged and older adults and is expected to increase in prevalence with the global population aging. Radiographic KOA affects more than 50% of people aged over 60 globally, and 20–30% are clinically affected (3). In addition to its impact on function and quality of life, KOA also imposes economic costs on patients, families, and society (4).

Conservatism is still the first-line treatment for KOA, usually involving pharmacotherapy (e.g., non-steroidal anti-inflammatory drugs, glucosamine, and intra-articular sodium hyaluronate), physical therapy, and in some cases surgical options (5), but long-term use of NSAIDs can lead to gastrointestinal and cardiovascular issues, and surgical procedures are expensive and potentially risky (6). These limitations have increased interest in safe, effective, and affordable non-pharmacological options (7).

Mongolian medicines warm acupuncture is a traditional external treatment of Mongolian medicine. It is performed by inserting specially designed silver needles into selected areas while heating the area using moxibustion or a warming device. The heating can penetrate deeper tissues. The method is believed to warm the meridians, promote He Yi flow and Qisu flow, relieve pain and inflammation, and harmonize the three roots—He Yi, Xila, and Badagan (8). In Mongolian medicine, KOA is referred to as joint Xieriwusu disease, which is attributed to excessive accumulation of Xieriwusu and Badagan/Qisu in the joint cavity, which can inhibit Heyi-Qisu flow. Warm acupuncture is believed (9) to relieve symptoms by dispelling cold and dampness and drying Xieriwusu.

Many RCTs have studied Mongolian medicines warm acupuncture for KOA in recent years, but the results are inconsistent, and sample sizes are small, which limits the certainty of the conclusions. To address these gaps, we conducted a systematic review and meta-analysis according to the Cochrane methodology, including subgroup and sensitivity analyses, an assessment of publication bias, and the GRADE approach to rate the certainty of the evidence. Our protocol was registered prospectively in PROSPERO (CRD420261362311), and we aimed to provide higher-level evidence to inform clinical decision-making.

## 2 Materials and methods

### 2.1 Protocol registration

This review was conducted and reported in accordance with the Preferred Reporting Items for Systematic Reviews and Meta-Analyses (PRISMA) 2020 statement (10, 11). The protocol was prospectively registered in PROSPERO under CRD420261362311 (12), with key methodological elements being prespecified to ensure transparency and reproducibility.

### 2.2 Information sources and search strategy

Eight databases were searched from their inception to 31 December 2025, including the China National Knowledge Infrastructure (CNKI), Wanfang Data, the Chinese Science and Technology Periodical Database (VIP), the Chinese Biomedical Literature Database (CBM), PubMed, Web of Science, the Cochrane Library, and Embase. The search strategies were tailored to each platform and combined controlled vocabulary (e.g., MeSH terms) with free-text keywords. Key Chinese concepts are written in pinyin with English glosses: menggu wen zhen (Mongolian medicines warm acupuncture), xigu guan jie yan (knee osteoarthritis), and sui ji dui zhao shi yan (randomized controlled trial). English terms include Mongolian medicines warm acupuncture, warm needling, knee osteoarthritis or gonarthrosis, and randomized controlled trial. Reference lists of the included studies and reviews were manually screened for additional eligible records. Full strategies for database strategies are summarized in the Supplementary material.

### 2.3 Eligibility criteria

Studies were eligible if they met all criteria within the Population, Intervention, Comparison, and Outcome (PICOS) framework.

#### 2.3.1 Participants

They included patients who were clinically diagnosed with KOA according to the American College of Rheumatology (ACR) clinical and radiographic criteria (or the Chinese Guidelines for Diagnosis and Treatment of Osteoarthritis [2018 edition], which are consistent with ACR standards). No restrictions were applied to age, sex, or disease severity.

#### 2.3.2 Intervention

It included Mongolian medicines warm acupuncture administered alone or alongside prespecified co-interventions (e.g., oral or topical Mongolian medicine and intra-articular sodium hyaluronate). As detailed above, the primary analysis was restricted to add-on designs, while head-to-head comparisons were analyzed separately as secondary analyses. Co-interventions were eligible only if applied consistently within and across the study arms; otherwise, the study was excluded from the primary analysis.

#### 2.3.3 Comparators

They included conventional Western medicine (e.g., glucosamine sulfate, NSAIDs, and intra-articular sodium hyaluronate), Mongolian medicine alone, or warm acupuncture alone.

Outcomes: The primary outcomes included clinical effective rate (total effective rate and binary classification). This outcome was defined in each included study as the proportion of patients achieving a predefined improvement threshold (typically ≥30% reduction in WOMAC score) or being rated as “excellent” or “good” according to the study’s own criteria (e.g., Mongolian medicine efficacy criteria, traditional Chinese medicine syndrome criteria, or Lysholm score). Despite variation in nomenclature, all definitions were based on composite measures of symptom and functional improvement and were treated as conceptually equivalent for the meta-analysis. All studies reporting this outcome used identical calculation methods (number of responders divided by total sample size × 100%). The secondary outcomes included pain intensity (visual analog scale, VAS), joint function (Western Ontario and McMaster Universities Osteoarthritis Index, WOMAC), and adverse events. When multiple time points were reported, end-of-treatment data were prioritized for analysis.

#### 2.3.4 Study design

This study included randomized controlled trials (RCTs) published in peer-reviewed journals or as dissertations.

#### 2.3.5 Exclusion criteria

The exclusion criteria included the following: non-RCT designs (quasi-experimental and observational), reviews, case reports, protocols, conference abstracts, or non-peer-reviewed sources; interventions not meeting the definition of warm acupuncture (e.g., acupuncture without needle heating or moxibustion alone); missing, incomplete, or non-extractable outcome data, or the use of outcome metrics incompatible with prespecified endpoints; duplicate publications or overlapping cohorts (the most comprehensive or recent report was retained); and multi-arm trials lacking an appropriate comparator or where co-interventions differed systematically between the arms.

### 2.4 Literature screening and data extraction

Two reviewers screened titles and abstracts, followed by full texts against eligibility criteria, and resolved disagreements through discussion or vote with a third reviewer. A pre-piloted extraction form included the first author, year, study setting, diagnostic criteria, sample size, baseline characteristics, intervention details (acupuncture procedure, warming procedure, frequency and duration of sessions, and co-interventions), comparator details, treatment duration, outcomes, time points, and adverse events. For dichotomous outcomes, the number of events and total participants were extracted; for continuous outcomes, means, standard deviations, and sample sizes were recorded. When multiple time points were reported, end-of-treatment data were prioritized.

### 2.5 Risk-of-Bias assessment

Risk of bias was assessed using the Cochrane risk of bias 2 (RoB 2.0) tool (13). In accordance with RoB 2 guidance, we assessed the risk of bias at the outcome level for the three primary outcomes: clinical response rate, pain (VAS), and joint function (WOMAC) (14). Two reviewers independently assessed five domains: (D1) randomization process, (D2) deviations from intended interventions, (D3) missing outcome data, (D4) measurement of the outcome, and (D5) selection of the reported result. Each domain was rated as low risk, some concerns, or high risk. Disagreements were resolved by discussion or consultation with a third reviewer. The assessments for the three outcomes yielded consistent results.

### 2.6 Statistical analysis

Meta-analyses were conducted using RevMan 5.4. For dichotomous outcomes (clinical response rate), pooled risk ratios (RRs) with 95% confidence intervals (CIs) were calculated using the Mantel–Haenszel method under a fixed-effects model. For continuous outcomes (VAS pain scores), mean differences (MDs) with 95% CIs were synthesized using inverse-variance weighting under a random-effects model with a DerSimonian–Laird estimator; the use of MDs was appropriate as all included studies used a 0–10 VAS with consistent units.

Between-study heterogeneity was assessed using Cochran’s Q test (*α* = 0.10) and quantified using the *I*^2^ statistic. *I*^2^ values of approximately 25, 50, and 75% were interpreted as low, moderate, and high heterogeneity, respectively; however, *I*^2^ > 50% was considered indicative of substantial heterogeneity.

Prespecified subgroup analyses for the primary outcome (clinical response rate) were planned based on treatment duration (≤2 weeks vs. >2 weeks) among the add-on design studies. Sensitivity analyses used a leave-one-out approach, iteratively omitting each study; studies exerting disproportionate influence were also temporarily excluded. Resulting pooled estimates, 95% CIs, and heterogeneity statistics were compared with primary analyses to assess robustness. Publication bias was explored by visual inspection of funnel plots when ≥10 studies were available; with fewer studies, funnel plots were not formally interpreted. All tests were two-sided, with a *p*-value of < 0.05 considered statistically significant.

### 2.7 Certainty of evidence

The GRADE approach was used to rate the certainty of evidence for the primary outcome (clinical response rate). Starting from high certainty for RCTs, the certainty of evidence was downgraded in five areas: risk of bias, inconsistency, indirectness, imprecision, and publication bias. Final ratings were categorized as high, moderate, low, or very low. Two reviewers made independent assessments, and disagreements were resolved through discussion or consultation with a third reviewer. All reasons for downgrading were clear and reported transparently.

## 3 Results

### 3.1 Study selection

**Figure 1:**
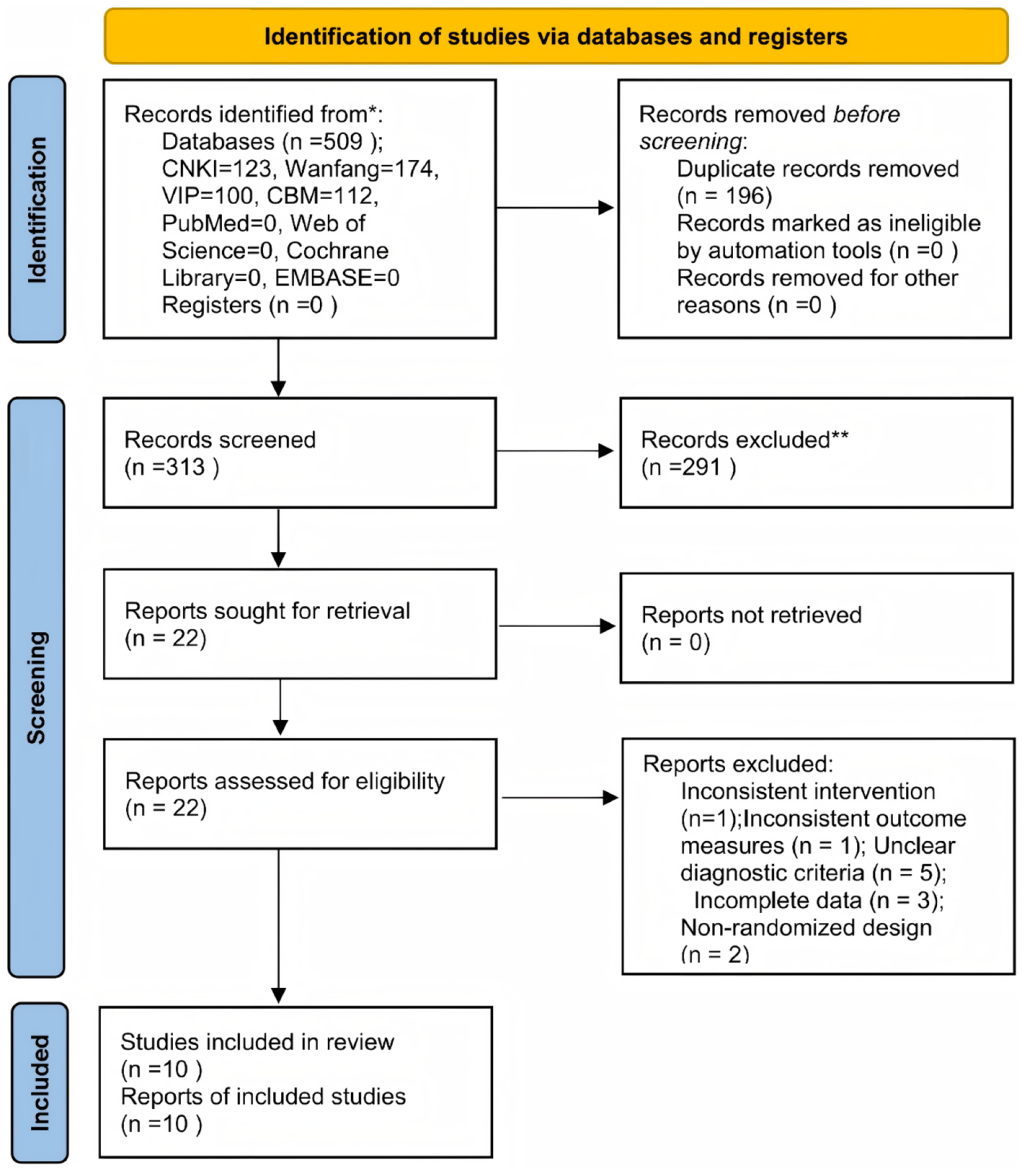
PRISMA 2020 flow diagram of study selection.

A total of 509 records were identified. After deduplication, 313 records remained. Title and abstract screening excluded 291 records, leaving 22 articles for full-text evaluation. Twelve were excluded for inconsistent interventions (*n* = 1), inconsistent outcome measures (*n* = 1), unclear diagnostic criteria (*n* = 5), incomplete data (*n* = 3), and non-randomized design (*n* = 2). Ten RCTs were included in the meta-analysis (15–24). Figure 1 shows the study selection process.

### 3.2 Characteristics of the included studies

The 10 studies were published between 2013 and 2023, all in Chinese, including nine articles and one thesis. A total of 882 participants were included. Trial sample sizes ranged from 60 to 124. All studies used a parallel-group design. Experimental groups received Mongolian medicines warm acupuncture alone (six trials) or combined with other therapies (four trials); control groups received conventional Western medicine, Mongolian medicine alone, warm acupuncture alone, or sodium hyaluronate. Treatment durations ranged from 2 to 5 weeks. The clinical response rate was reported in nine studies, the VAS score in three studies, and the WOMAC score in four studies. Study characteristics are summarized in Table 1.

**Table 1:** Characteristics of the included studies.

Study	Sample size (E/C)	Experimental intervention	Control intervention	Duration	Outcomes
Wu Terigen 2023 (15)	30/30	Mongolian medicines warm acupuncture + sodium hyaluronate	Sodium hyaluronate alone	4 weeks	①②③④
Nie Yulin 2013 (22)	35/35	Mongolian medicines warm acupuncture + sodium hyaluronate	Mongolian medicines warm acupuncture alone	5 weeks	②⑤
Chaoke Bilige 2015 (21)	30/30	Mongolian medicines warm acupuncture + oral Mongolian herb	Oral Mongolian acupuncture herb alone	1 month	①②
Chen Ying and Alateng Qimuge (2017) (20)	31/31	Mongolian medicines warm acupuncture + topical Mongolian herb	Mongolian medicines warm acupuncture alone	2 weeks	①
Aliguna 2021 (17)	53/53	Mongolian medicines warm acupuncture alone	Oral glucosamine sulfate	2 weeks	①③
Xue Feng 2018 (18)	62/62	Mongolian medicines warm acupuncture alone	Oral glucosamine sulfate	1 month	①④
Morigen 2023 (16)	56/56	Mongolian medicines warm acupuncture alone	Oral loxoprofen sodium	4 weeks	①③④
Bai Xiaohong (2018) (19)	48/48	Mongolian medicines warm acupuncture alone	Oral glucosamine sulfate	1 month	①③
Aruheng 2022 (23)	50/50	Mongolian medicines warm acupuncture alone	Oral glucosamine sulfate	2 weeks	①
Wang Qiming and Bao Xiaohong (2018) (24)	46/46	Mongolian medicines warm acupuncture alone	Oral glucosamine hydrochloride + celecoxib	1 month	①④

E/C, experimental/control group sample size. Outcomes: ① clinical effective rate; ② VAS score; ③ WOMAC score; ④ knee function score; ⑤ Lysholm score.

### 3.3 Risk of Bias

**Figure 2:**
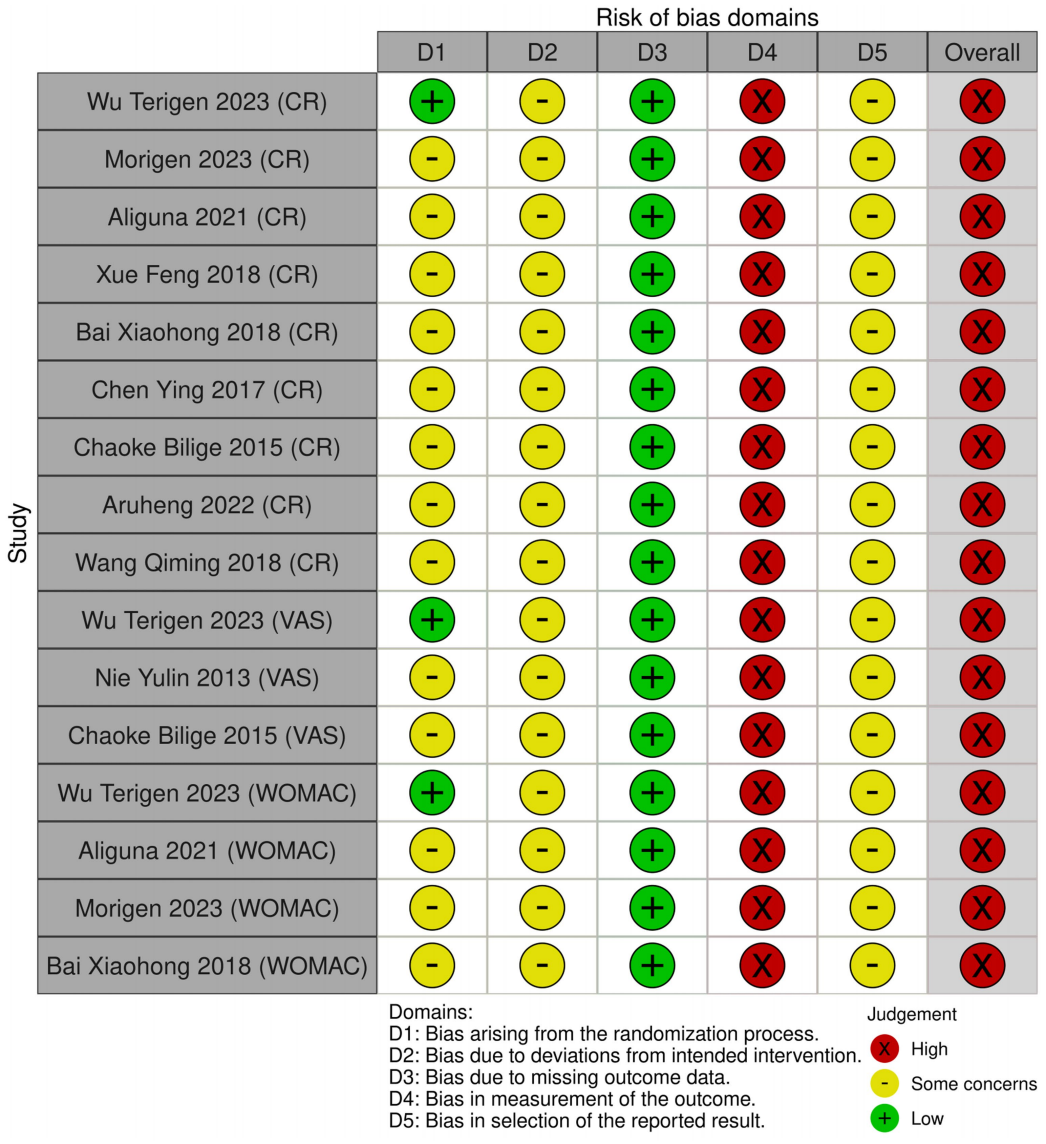
Risk of bias traffic light plot for the primary outcome.

**Figure 3:**
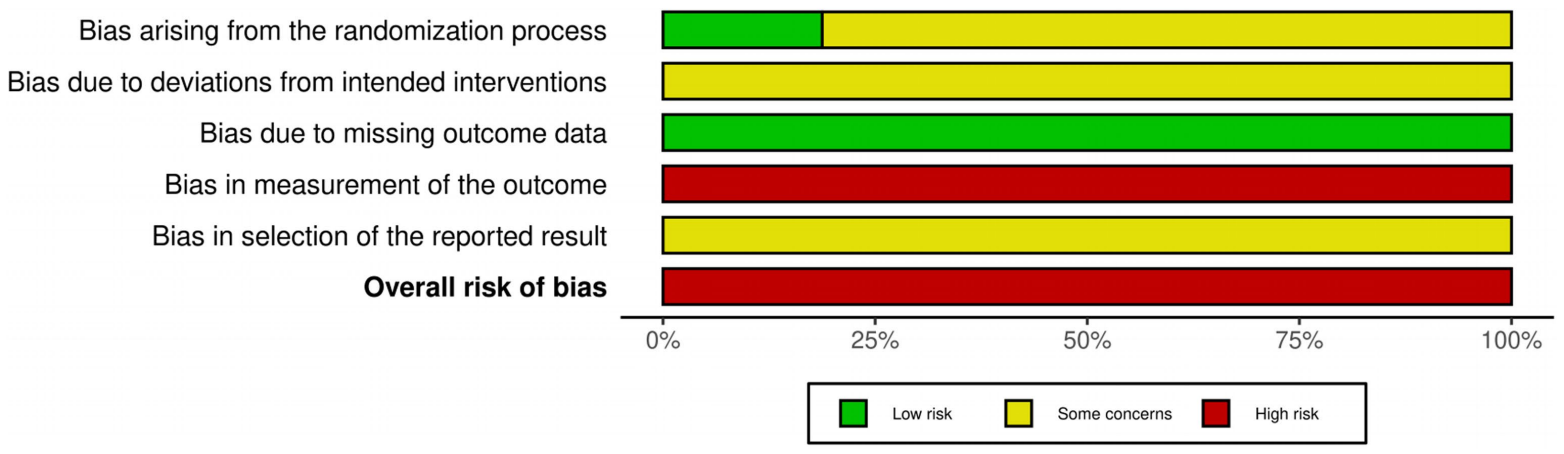
Risk of bias summary bar plot.

The risk of bias was assessed at the outcome level for clinical response rate (CR), VAS, and WOMAC. The assessments for the three outcomes yielded consistent results. The traffic light plot for clinical response rate is presented as a representative summary in Figures 2, 3, with identical ratings applying to VAS and WOMAC. For D1 (the randomization process), one study (15) was rated as low risk (random number table generation and allocation concealment using sealed envelopes), while the remaining eight studies were rated as having some concerns (randomization stated but methods not detailed). For D2 (deviations from intended interventions), all nine studies were rated as having some concerns (blinding of participants and therapists was not feasible). For D3 (missing outcome data), all nine studies were rated as low risk (no dropouts reported). For D4 (measurement of the outcome), all nine studies were rated as high risk (lack of assessor blinding for the subjective outcomes, including VAS and WOMAC). For D5 (selection of the reported result), all nine studies were rated as having some concerns (no prospectively registered protocol). Overall, all nine studies contributing to the clinical response rate outcome were judged to have a high risk of bias.

### 3.4 Clinical response rate

#### 3.4.1 Primary meta-analysis: add-on design

**Figure 4:**
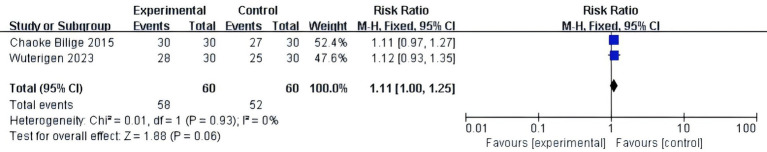
Forest plot of clinical response rate in add‑on design studies (fixed‑effect model).

Two studies (15, 21) strictly adhered to the add-on design, in which the experimental group received Mongolian medicines warm acupuncture in addition to the same intervention as the control group, while the control group received that intervention alone. Between-study heterogeneity was negligible (*I*^2^ = 0%, *p* = 0.93), and a fixed-effects model was used. The pooled clinical response rate showed a non-significant benefit of Mongolian medicines warm acupuncture (RR = 1.11, 95% CI: 1.00–1.25; *p* = 0.06; Figure 4).

#### 3.4.2 Secondary meta-analysis: warm acupuncture alone and oral medications

**Figure 5:**
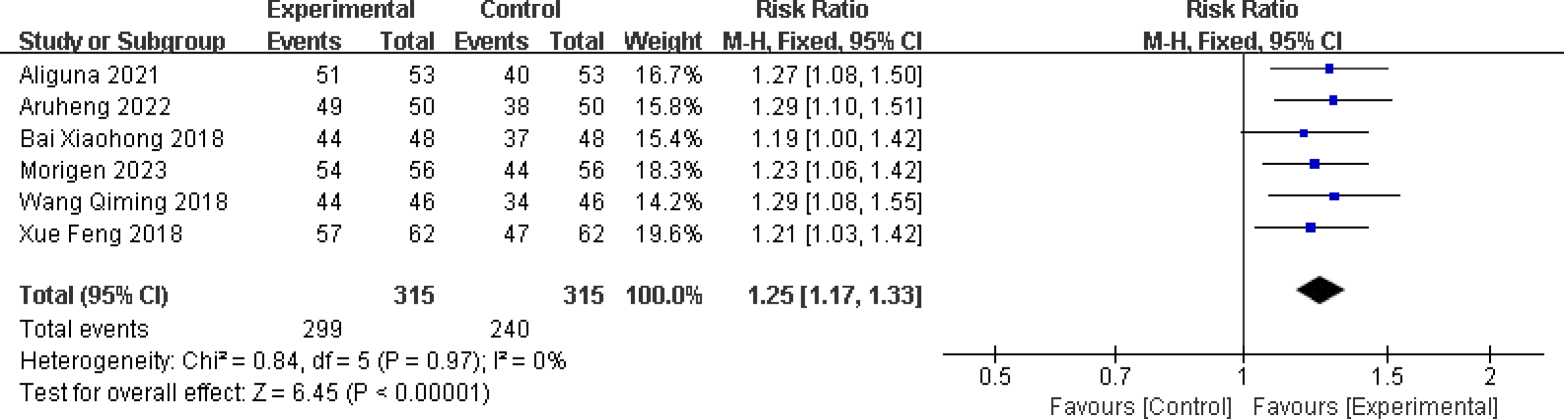
Forest plot of clinical response rate in head‑to‑head comparisons (warm acupuncture alone vs. oral medications, fixed‑effect model).

Six studies compared Mongolian medicines warm acupuncture alone with oral medications (glucosamine sulfate, loxoprofen sodium, or celecoxib). Between-study heterogeneity was negligible (*I*^2^ = 0%, *p* = 0.97), and a fixed-effects model was used. Mongolian medicines warm acupuncture significantly improved the clinical response rate compared with oral medications (pooled RR = 1.25, 95% CI: 1.17–1.33; *p* < 0.00001; Figure 5).

**Figure 6:**
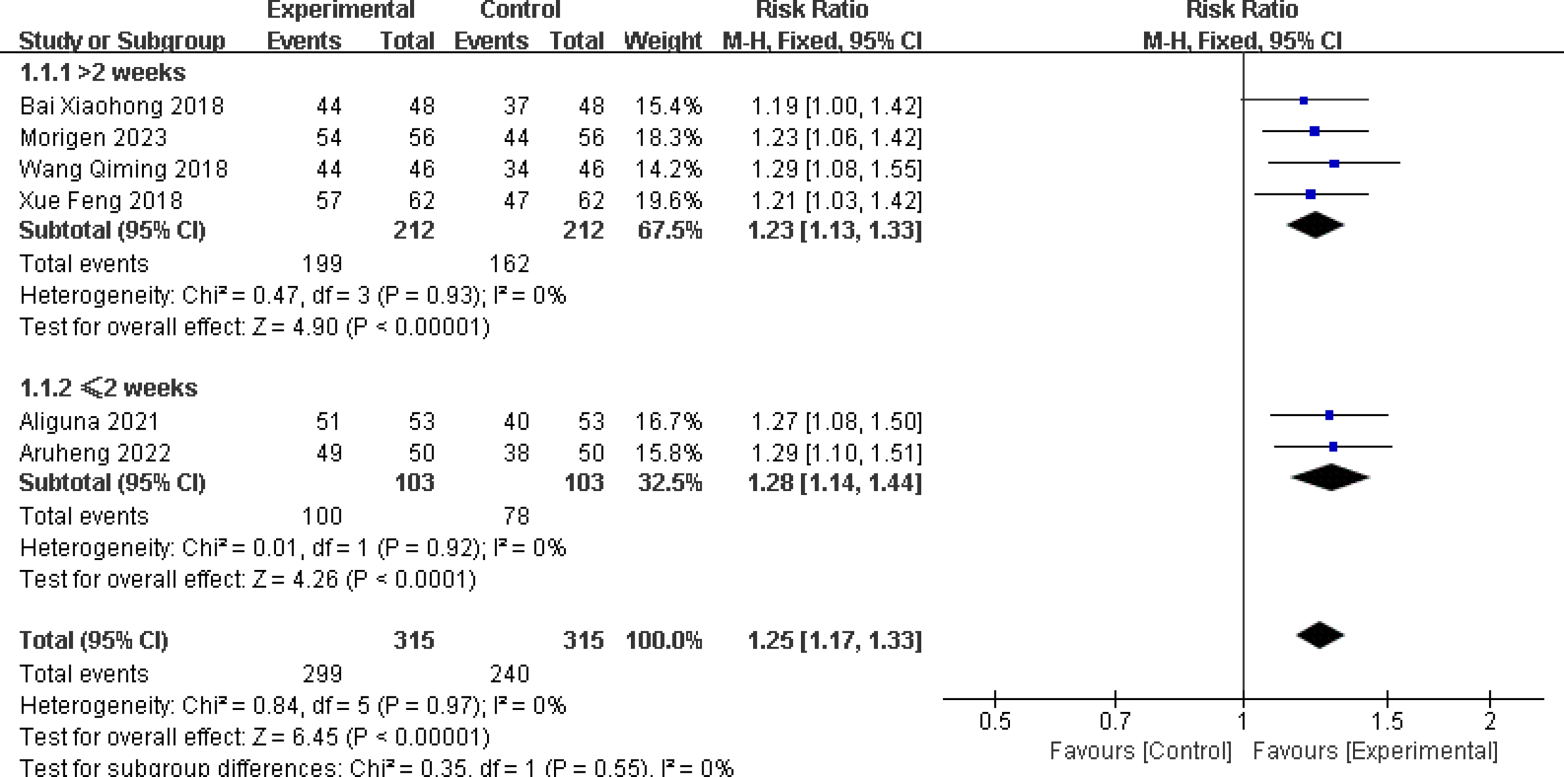
Subgroup analysis by treatment duration (≤2 weeks vs. >2 weeks) for clinical response rate in head‑to‑head comparisons (fixed‑effect model).

Among the six head-to-head studies, we performed a subgroup analysis according to treatment duration (≤2 weeks vs. >2 weeks). Two studies (17, 23) had a duration of 2 weeks (pooled RR = 1.28, 95% CI: 1.14–1.44; *p* < 0.0001; *I*^2^ = 0%), and four studies (16, 18, 19, 24) had durations of 3–4 weeks (pooled RR = 1.23, 95% CI: 1.13–1.33; *p* < 0.00001; *I*^2^ = 0%). The test for subgroup difference was not significant (Chi^2^ = 0.35, df = 1, *p* = 0.55; *I*^2^ = 0%), indicating that the treatment effect did not differ significantly by treatment duration (Figure 6).

### 3.5 Pain score (VAS)

**Figure 7:**
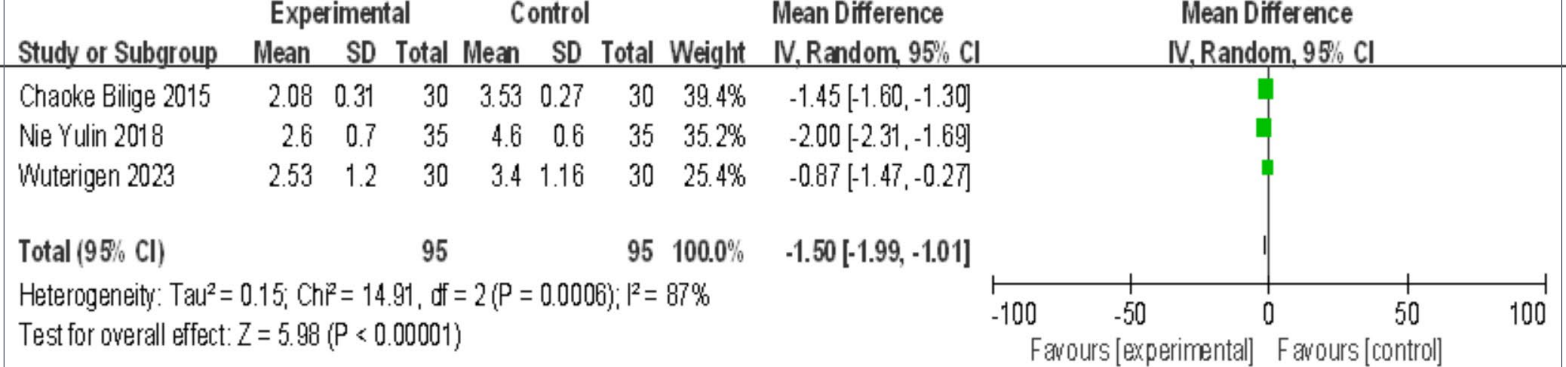
Forest plot of VAS score (random‑effects model).

Three studies reported post-treatment VAS scores (0–10 scale). Substantial heterogeneity was detected (*I*^2^ = 87%, *p* = 0.0006), and a random-effects model was used. Mongolian medicines warm acupuncture significantly reduced pain compared with the control intervention (pooled MD = −1.50, 95% CI: −1.99 to −1.01; *p <* 0.00001; Figure 7). Given the small number of contributing studies, this result should be interpreted cautiously.

### 3.6 Joint function (WOMAC)

**Figure 8:**
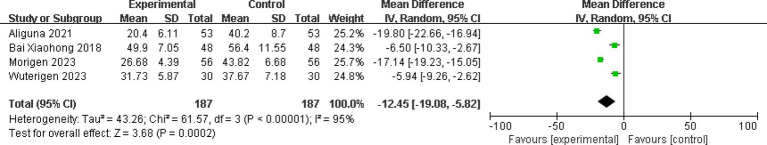
Forest plot of WOMAC total score (random‑effects model).

Four studies reported post-treatment WOMAC total scores. Substantial heterogeneity was detected (*I*^2^ = 95%, *p* < 0.00001), and a random-effects model was used. Mongolian medicines warm acupuncture significantly improved joint function compared with controls (pooled MD = −12.45, 95% CI: −19.08 to −5.82; *p* = 0.0002; Figure 8). Given the substantial heterogeneity and the small number of studies, this result should be interpreted cautiously.

### 3.7 Sensitivity analysis

**Figure 9:**
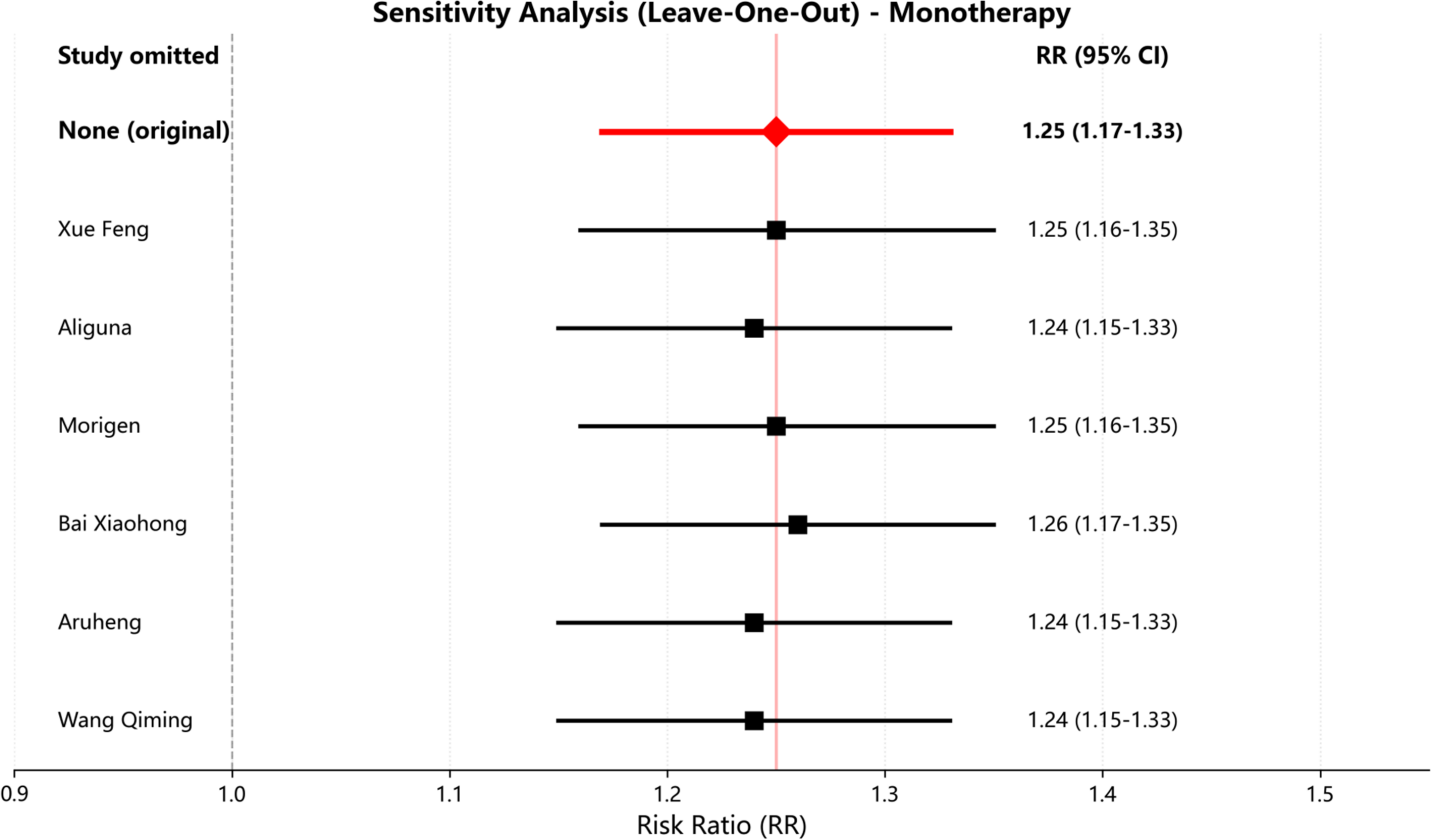
Sensitivity analysis for monotherapy.

Leave-one-out sensitivity analysis demonstrated the robustness of the pooled estimates for the secondary meta-analysis (six head-to-head studies). After sequentially excluding each study, the pooled RR ranged from 1.24 to 1.26, the lower 95% CI limit remained above 1.0, *I*^2^ was consistently 0%, and the *p-*value was always < 0.00001, indicating that no single study unduly influenced the results (Figure 9).

### 3.8 Publication bias

**Figure 10:**
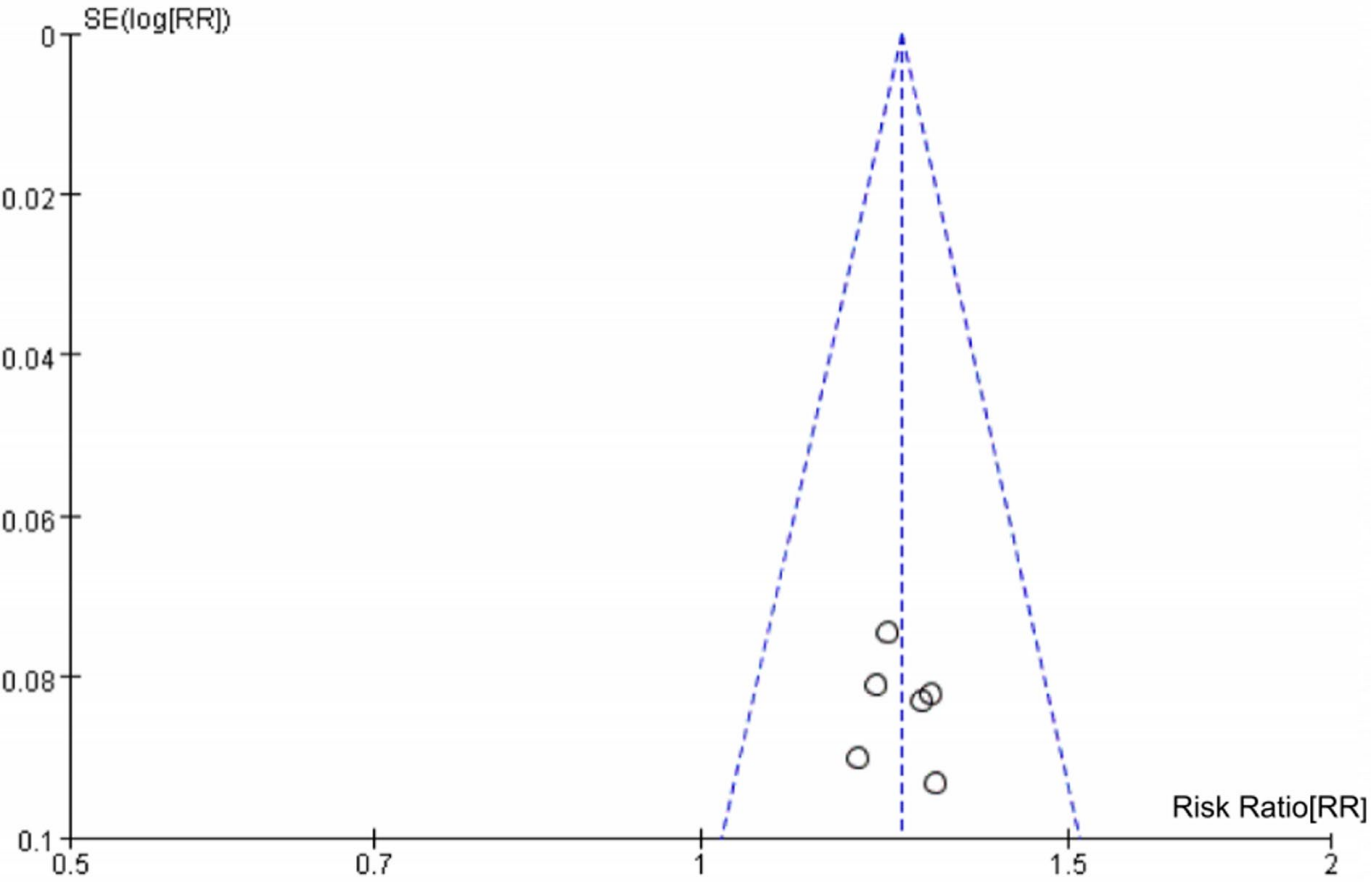
Funnel plot for publication bias of clinical response rate (secondary meta‑analysis, six head‑to‑head studies).

We derived a funnel plot for the secondary meta-analysis (six head-to-head studies) to assess publication bias (Figure 10). The data were distributed around the pooled RR of 1.25, with no obvious asymmetry. However, this should be interpreted with caution, as the limited number of studies (*n* = 6) does not provide sufficient power to formally assess publication bias (see Table 2).

**Table 2:** Leave-one-out sensitivity analysis for the secondary meta-analysis.

Study excluded	Pooled RR	95% CI	*I*^2^ (%)	*p* value	Robust
None (original)	1.25	1.17–1.33	0	<0.00001	Yes
Aliguna 2021 (17)	1.24	1.15–1.33	0	<0.00001	Yes
Aruheng 2022 (23)	1.24	1.15–1.33	0	<0.00001	Yes
Bai Xiaohong (2018) (19)	1.26	1.17–1.35	0	<0.00001	Yes
Morigen 2023 (16)	1.25	1.16–1.35	5	<0.00001	Yes
Wang Qiming and Bao Xiaohong (2018) (24)	1.24	1.15–1.33	0	<0.00001	Yes
Xue Feng 2018 (18)	1.25	1.16–1.35	0	<0.00001	Yes

RR, risk ratio; CI, confidence interval. Robustness was defined as the pooled RR remaining statistically significant (*p* < 0.00001), with the lower limit of the 95% CI > 1.0 after exclusion of each study.

### 3.9 Certainty of evidence

The certainty of evidence was assessed using the GRADE approach (Table 3). For the primary analysis (add-on design, two studies), the certainty was downgraded to low (⊕ ⊕ ○○) due to serious risk of bias (lack of assessor blinding) and very serious imprecision (wide confidence interval crossing the null value and small sample size).

**Table 3:** GRADE evidence profile for the main outcomes.

Outcome	Number of studies (participants)	Relative effect (95% CI)	Absolute effect	Quality of evidence (GRADE)	Reasons for downgrading
Primary analysis: add-on design	2 studies (*n* = 120)	RR 1.11 (1.00–1.25)	Control: 867/1000; experimental: 962/1000 (95 more)	⊕ ⊕ ○○ Low	Risk of bias (serious: lack of assessor blinding); imprecision (very serious: CI crosses 1.0, small sample size)
Secondary analysis: head-to-head comparisons	6 studies (*n* = 630)	RR 1.25 (1.17–1.33)	Control: 762/1000; experimental: 952/1000 (190 more)	⊕ ⊕ ⊕○ Moderate	Risk of bias (serious: lack of assessor blinding); no downgrading for inconsistency (*I*^2^ = 0%), indirectness, or imprecision
VAS pain score	3 studies (*n* = 190)	MD –1.50 (−1.99 –1.01)	—	⊕ ⊕ ○○ Low	Risk of bias (serious: lack of assessor blinding); inconsistency (serious: *I*^2^ = 87%)
WOMAC total score	4 studies (*n* = 374)	MD –12.45 (−19.08 –5.82)	—	⊕ ⊕ ○○ Low	Risk of bias (serious: lack of assessor blinding); Inconsistency (serious: *I*^2^ = 95%)

RR, risk ratio; MD, mean difference; CI, confidence interval. GRADE ratings were based on the following criteria: risk of bias: all studies lacked assessor blinding (serious). Inconsistency: downgraded when *I*^2^ > 50% (VAS: 87%; WOMAC: 95%). Imprecision: downgraded for add-on analysis due to a wide CI crossing 1.0 and a small sample size. Indirectness and publication bias were not downgraded for any outcome. Control event rates for dichotomous outcomes: add-on analysis (52/60 = 86.7%); head-to-head analysis (240/315 = 76.2%). Absolute effect = control event rate × RR. GRADE quality ratings: high (⨁⨁⨁⨁), moderate (⨁⨁⨁○), low (⨁⨁○○), and very low (⨁○○○).

For the secondary analysis (head-to-head comparisons, six studies), the certainty was rated as moderate (⊕ ⊕ ⊕○), downgraded one level due to serious risk of bias (lack of assessor blinding). No downgrading was applied for inconsistency (*I*^2^ = 0%), indirectness, or imprecision (precise effect estimate with a narrow confidence interval). For the VAS outcome (three studies), certainty was downgraded to low (⊕ ⊕ ○○) due to serious risk of bias (lack of assessor blinding) and serious inconsistency (*I*^2^ = 87%).

For the WOMAC outcome (four studies), certainty was downgraded to low (⊕ ⊕ ○○) due to serious risk of bias (lack of assessor blinding) and very serious inconsistency (*I*^2^ = 95%).

### 3.10 Adverse events

None of the 10 included studies reported serious adverse effects associated with Mongolian medicines warm acupuncture. Only two studies reported no obvious adverse effects during treatment, and none collected or reported safety data systematically. We therefore did not perform a quantitative meta-analysis of Mongolian medicines warm acupuncture safety.

## 4 Discussion

### 4.1 Main findings

This systematic review evaluated the efficacy and safety of Mongolian medicines warm acupuncture for KOA across 10 RCTs (*n* = 882). Key findings are as follows:

(1) Clinical response rate: For the primary analysis (add-on design), two studies (*n* = 120) showed a non-significant benefit of Mongolian medicines warm acupuncture (RR = 1.11, 95% CI: 1.00–1.25; *p* = 0.06; *I*^2^ = 0%). For the secondary analysis (head-to-head comparisons), six studies (*n* = 630) showed that Mongolian medicines warm acupuncture alone significantly improved clinical response compared with oral medications (RR = 1.25, 95% CI: 1.17–1.33; *p* < 0.00001; *I*^2^ = 0%). Subgroup analysis by treatment duration (≤2 weeks vs. >2 weeks) within the head-to-head studies showed no significant difference between the subgroups (*P* for subgroup difference = 0.55), suggesting that the treatment effect did not vary substantially by treatment duration.

(2) Pain relief (VAS): Three trials reported post-treatment VAS scores. A random-effects model was used due to substantial heterogeneity (*I*^2^ = 87%). Mongolian medicines warm acupuncture significantly reduced pain compared with the control intervention (pooled MD = −1.50, 95% CI: −1.99 to −1.01; *p* < 0.00001). The high heterogeneity—likely reflecting differences in control interventions, treatment frequency, and follow-up time points—reduces confidence in this estimate.

(3) Joint function (WOMAC): Four trials reported post-treatment WOMAC total scores. A random-effects model was used due to substantial heterogeneity (*I*^2^ = 95%). Mongolian medicines warm acupuncture significantly improved joint function compared with the control intervention (pooled MD = −12.45, 95% CI: −19.08 to −5.82; *p* = 0.0002). Given the substantial heterogeneity and the small number of studies, this result should be interpreted cautiously.

(4) Certainty of evidence: The certainty of evidence for the secondary analysis (head-to-head comparisons) was rated as moderate, while the primary analysis (add-on design), VAS, and WOMAC outcomes were rated as low due to risk of bias, imprecision, or substantial heterogeneity.

### 4.2 Comparison with previous systematic reviews

To the best of our knowledge, this is the first systematic review and meta-analysis specifically evaluating Mongolian medicines warm acupuncture for KOA. Previous meta-analyses have examined acupuncture for KOA more broadly, and an overview of systematic reviews by Jun et al. (25) found that warm needle acupuncture was effective for osteoarthritis but noted that the methodological quality of existing reviews was critically low. Our study was conducted in accordance with the PRISMA 2020 guidelines, with the protocol prospectively registered in PROSPERO, using the RoB 2 tool for bias assessment and the GRADE approach for evidence certainty rating. These methodological features are consistent with current best practices and enhance the transparency and reproducibility of our findings. Our results are consistent with the general evidence base for warm acupuncture while extending it specifically to Mongolian medicines warm acupuncture and providing more granular analyses based on study design and treatment duration.

### 4.3 Potential mechanisms of acupuncture in KOA

Emerging evidence has suggested that acupuncture may exert therapeutic effects in KOA through multiple interconnected pathways. A network meta-analysis by Liu et al. (26) found that warm acupuncture can reduce pro-inflammatory cytokines (interleukin-1β [IL-1β], IL-6, and tumor necrosis factor-alpha [TNF-*α*]) and modulate the endogenous opioid system, providing both anti-inflammatory and analgesic effects. At the molecular level, acupuncture-related therapy has been shown to regulate immune-inflammatory pathways, which may be mediated through Damage-associated molecular patterns (DAMPs)/Toll-like receptor (TLR)/Nuclear factor-kappa B (NF-κB)/Mitogen-activated protein kinase (MAPK), Phosphatidylinositol 3-kinase (PI3K)/Protein kinase B (Akt)/NF-κB or Interferon-gamma (IFN-γ)/Janus kinase (JAK)-Signal transducer and activator of transcription (STAT) signaling cascades. Additionally, acupuncture may inhibit cytokine expression in the p38MAPK pathway to attenuate cartilage extracellular matrix degradation, regulate mitochondrial pathway cytokines to inhibit chondrocyte apoptosis, and delay cartilage degeneration. A recent systematic review by Zhang et al. (27) on acupuncture for pregnancy-related pain further supported the role of acupuncture in central pain modulation. Similarly, Garcia-Espinosa et al. (28) demonstrated that acupuncture induces gene co-expression networks involved in inflammation and bone metabolism in postmenopausal women with osteoarthritis, providing transcriptomic evidence supporting its therapeutic effects in osteoarthritis.

For Mongolian medicines warm acupuncture, the added thermal stimulation may offer additional benefits beyond conventional acupuncture. Warm needling has been shown to significantly improve local blood flow and oxygen delivery capacity in KOA patients, with these improvements persisting longer than those achieved by needling without heat. The heat component is believed to promote local vasodilation, improve microcirculation, and “dispel cold and dampness,” as conceptualized in Mongolian medicine, which may enhance the clearance of inflammatory mediators and accelerate tissue repair. These mechanistic insights—encompassing anti-inflammation, central pain modulation, cartilage protection, and local hemodynamic improvement—provide biological plausibility for the clinical effects observed in our meta-analysis (28).

### 4.4 Clinical implications

1. Non-pharmacological option for pain relief: For KOA patients who cannot tolerate or prefer to avoid NSAIDs, Mongolian medicines warm acupuncture represents a feasible, low-cost alternative. The technique may be particularly suitable for primary care settings and regions where Mongolian medicine is practiced. Although adverse events were not systematically reported, no serious adverse effects were documented in the included trials, suggesting that the treatment is generally well tolerated in the available evidence.
2. Add-on versus standalone use: In add-on design studies (warm acupuncture plus standard care vs. standard care alone), the benefit did not reach statistical significance (RR = 1.11, 95% CI: 1.00–1.25; *p* = 0.06), likely due to the small number of trials and limited sample size. In contrast, head-to-head comparisons of warm acupuncture alone versus oral medications showed a significant and robust benefit (RR = 1.25, 95% CI: 1.17–1.33; *p* < 0.00001). This suggests that Mongolian medicines warm acupuncture may be a viable alternative to conventional oral analgesics for patients who prefer non-pharmacological treatment or who have contraindications to these medications.
3. Number needed to treat (NNT): Based on the secondary analysis (head-to-head comparisons), the control group efficacy rate was 76.2%, with an absolute risk difference of approximately 18.8%, corresponding to an NNT of 5. This indicates that approximately five patients would need to be treated with Mongolian medicines warm acupuncture to achieve one additional clinically favorable outcome compared with oral medication.
4. Accessibility and clinical applicability: Mongolian medicines warm acupuncture is low-cost, minimally invasive, and does not require expensive equipment. These characteristics make it particularly suitable for integration into primary care and community-based rehabilitation programs, particularly in ethnic minority regions where Mongolian medicine has greater cultural acceptability. However, implementation should be supported by adequate training of practitioners and further high-quality confirmatory trials.

### 4.5 Limitations

Methodological limitations: The overall risk of bias was high. Blinding of participants and personnel was generally infeasible, and the majority of trials lacked adequate descriptions of sequence generation or allocation concealment. Outcomes were frequently assessed by non-blinded clinicians or via patient self-report, increasing the risk of detection bias.

Clinical heterogeneity: Warm- acupuncture procedures varied across studies in acupoint selection, needling depth, the number of moxibustion cones, treatment frequency, and course length. Control interventions were also heterogeneous. Although subgroup analyses partially addressed these differences, residual confounding remains possible.

Statistical limitations: The add-on design primary analysis included only two studies with a wide confidence interval crossing the null, limiting statistical power and precision. The VAS meta-analysis included only three studies with high heterogeneity (*I*^2^ = 87%), reducing confidence in the pooled estimate. The WOMAC meta-analysis also showed substantial heterogeneity (*I*^2^ = 95%), likely due to differences in WOMAC versions and baseline severity across the studies.

Publication bias: With only six studies in the secondary analysis, the power to detect small-study effects was limited; a roughly symmetric funnel plot does not exclude publication bias or small-study effects.

Safety data: Adverse events were not systematically collected or reported in the majority of trials, precluding quantitative synthesis of harms. The majority of reports provided only qualitative statements (e.g., “no obvious adverse reactions”).

### 4.6 Recommendations for future research

1. Study design and reporting: Large, multicenter, prospectively registered RCTs adhering to the Consolidated Standards of Reporting Trials (CONSORT) guidelines are needed. Where participant blinding is impractical, outcome assessors should be blinded, and objective measures (e.g., imaging findings and serum inflammatory markers) should be incorporated.
2. Outcome standardization: The WOMAC total score (0–96) and a 0–10 VAS score should be adopted as core outcomes, with harmonized assessment time points (end of treatment, 3 months, and 6 months) to facilitate future pooling of data.
3. Intervention standardization: Detailed, reproducible descriptions of warm acupuncture—including acupoints, stimulation intensity, the number of moxibustion cones, session frequency, and the total treatment course—should be provided, along with a full specification of any co-interventions.
4. Safety monitoring: Adverse events should be systematically collected and reported, including type, incidence, and severity, to enable future meta-analyses of safety outcomes.
5. Comparative effectiveness: Head-to-head trials comparing Mongolian medicines warm acupuncture with other traditional medical practices (e.g., Tibetan or Uyghur medicine) and contemporary rehabilitation modalities (e.g., physical therapy and structured exercise) are needed to clarify their relative benefits.

## 5 Conclusion

Moderate-certainty studies have suggested that Mongolian medicines warm acupuncture improves clinical effectiveness and reduces pain in KOA. The results from the subgroups suggest that Mongolian medicines warm acupuncture alone may outperform combined therapy, although this is based on a few low-certainty studies. Because Since the risk of bias is high and the clinical heterogeneity is substantial, these results should be taken with caution. Well-designed RCTs with standard protocols and outcome measures are needed to verify long-term effectiveness and safety.

## Data Availability

Publicly available datasets were analyzed in this study. This data can be found here: this study is a systematic review and meta-analysis. All data are derived from previously published studies and are fully presented in the manuscript’s tables and figures. No original dataset was generated. Further inquiries can be directed to the corresponding author.
