# Supplementary material for: Efficacy of Mongolian medicines warm acupuncture for knee osteoarthritis: a systematic review and meta-analysis
Source: Front Med (Lausanne). 2026 Jul 22;13:1860943. doi: 10.3389/fmed.2026.1860943 (PMC13437308; doi:10.3389/fmed.2026.1860943)
Supplement: Supplementary file 1 [file Data_Sheet_1.PDF]

Supplementary Material

Search Strategies

1.CNKI（中国知网）

(SU='蒙医温针' OR SU='蒙医温针灸' OR SU='温针' OR SU='温针灸') AND (SU='膝骨关节炎' OR SU='膝关节骨性关节炎' OR SU='KOA' OR SU='膝痹') AND (SU='随机' OR SU='随机对照' OR SU='RCT')

2. Wanfang（万方）

主题:(蒙医温针 OR 蒙医温针灸 OR 温针 OR 温针灸) AND 主题:(膝骨关节炎 OR 膝关节骨性关节炎 OR KOA OR 膝痹) AND 主题:(随机 OR 随机对照 OR RCT)

3. VIP（维普）

(M=蒙医温针 OR M=蒙医温针灸 OR M=温针 OR M=温针灸) AND (M=膝骨关节炎 OR M=膝关节骨性关节炎 OR M=KOA OR M=膝痹) AND (M=随机 OR M=随机对照 OR M=RCT)

4. CBM（中国生物医学文献数据库）

(蒙医温针 OR 蒙医温针灸 OR 温针 OR 温针灸) AND (膝骨关节炎 OR 膝关节骨性关节炎 OR KOA OR 膝痹) AND (随机 OR 随机对照 OR RCT)

5. PubMed

("Mongolian warm acupuncture" OR "warm needling" OR "warm acupuncture" OR "Mongolian acupuncture") AND ("knee osteoarthritis" OR "gonarthrosis" OR "knee OA" OR "osteoarthritis, knee"[MeSH]) AND ("randomized controlled trial"[pt] OR "controlled clinical trial"[pt] OR "randomized"[tiab] OR "placebo"[tiab] OR "RCT"[tiab])

6. Web of Science

TS=("Mongolian warm acupuncture" OR "warm needling" OR "warm acupuncture" OR "Mongolian acupuncture") AND TS=("knee osteoarthritis" OR "gonarthrosis" OR "knee OA") AND TS=("randomized" OR "randomised" OR "RCT" OR "controlled trial")

7. Cochrane Library

("Mongolian warm acupuncture" OR "warm needling" OR "warm acupuncture") AND ("knee osteoarthritis" OR "gonarthrosis") AND ("randomized controlled trial" OR "RCT")

8. EMBASE

('Mongolian warm acupuncture'/exp OR 'Mongolian warm acupuncture' OR 'warm needling' OR 'warm acupuncture') AND ('knee osteoarthritis'/exp OR 'knee osteoarthritis' OR 'gonarthrosis') AND ('randomized controlled trial'/exp OR 'randomized controlled trial' OR 'RCT')

Note: The search strategies were adjusted to meet the specific syntax requirements of each database. All searches were conducted from inception to December 31, 2025. No language or publication type restrictions were applied, except where database filters were required.
